# Influence of prone, supine, and lateral positions during spine surgery on vascular, abdominal, and postural anatomy: a comprehensive review and Bayesian meta-analysis

**DOI:** 10.1186/s40001-025-03239-2

**Published:** 2025-10-07

**Authors:** Samir Smajic, Markus Konieczny, Koroush Kabir, Raffaele Scrofani, Filippo Migliorini, Anel Dracic

**Affiliations:** 1 Department of Spine Surgery, Academic Hospital of Düren, Düren, Germany; 2 University of Witten/ Herdecke, Witten, Germany; 3 Orthopädische Klinik Volmarstein, Klinik für Wirbelsäulenchirurgie, Wetter, Germany; 4 Helios Klinik Wuppertal, Klinik für Unfallchirurgie, Orthopädie Und Sportmedizin, Wuppertal, Germany; 5 Department of Trauma and Reconstructive Surgery, University Hospital of Halle, Martin-Luther University Halle-Wittenberg, Ernst-Grube-Street 40, 06097 Halle (Saale), Germany; 6 Department of Orthopaedic and Trauma Surgery, Academic Hospital of Bolzano (SABES-ASDAA), 39100 Bolzano, Italy; 7 Department of Orthopaedic and Trauma Surgery, Health, and Health Professions, Link Campus University, Rome, Italy; 8 Department of Neurosurgery, University of Salerno, Salerno, Italy

**Keywords:** Lumbar spine, Patient positioning, Prone position, Lateral decubitus, Vascular displacement, Retroperitoneal organs, Lordosis, Meta‑analysis

## Abstract

**Background:**

Patient positioning alters the three-dimensional relationship between the spine and surrounding neurovascular and visceral structures, thereby influencing both the technical feasibility and safety of lumbar procedures. Quantitative estimates of these positional shifts remain heterogeneous.

**Objective:**

To determine, across contemporary imaging studies, how prone, supine, and lateral decubitus positions alter the displacement of great vessels and retroperitoneal organs, the location of the psoas/lumbar plexus, and segmental lumbar lordosis.

**Methods:**

MEDLINE, Embase, and CENTRAL were searched from 2015 to 2025. Eligible studies compared at least two positions in adults and reported millimetre or degree differences for the outcomes of interest. Random‑effects (REML) subgroup meta‑analyses, a graph‑theoretical network meta‑analysis (netmeta), leave‑one‑out diagnostics, and Bayesian sensitivity models were performed. Risk of bias was assessed with ROBINS‑I.

**Results:**

Nine studies (41 independent comparisons; *n* = 1,248) met inclusion criteria. Retro‑peritoneal organs moved posteriorly by a pooled + 6.34 mm (95% CI  1.87–10.80; *p* = 0.007) when patients were turned from lateral decubitus to the prone position, narrowing the anterior working corridor at L2–L4. No significant pooled displacement was detected for major vessels (+ 1.26 mm, 95% CI −2.43–4.94), psoas/plexus (+ 0.94 mm, 95% CI −3.58–5.46) or segmental lordosis (+ 1.55°, 95% CI −4.62–7.73°). Direct contrasts showed that the supine-to-prone transition increased combined displacement/lordosis by + 3.64 mm / °(95% CI 0.53–6.76). Network ranking favoured the supine position for anatomical stability, but inconsistency was high (*I*^2^ = 89%). Two studies were low, three moderate, three serious and one critical risk of bias; removing serious/critical studies did not change the effect direction.

**Conclusions:**

Turning a patient prone produces a reproducible posterior migration of the colon and kidney (6 mm) and a modest increase in lumbar lordosis (3–4°). Vascular and psoas positions are highly patient-specific and cannot be assumed based on supine imaging alone. Preoperative planning should therefore incorporate position-matched imaging or intraoperative navigation, especially for anterior or anterolateral approaches at L2–L4. Further high-quality, multi-positional imaging studies are warranted to clarify the sources of the marked heterogeneity observed.

**Supplementary Information:**

The online version contains supplementary material available at 10.1186/s40001-025-03239-2.

## Introduction

The surgical positioning of patients during spine surgery is a crucial determinant of the procedure’s success [1–10]. Different positions (prone, supine, and lateral) offer unique benefits and challenges, particularly in relation to the anatomy of vascular structures, abdominal contents, and the musculoskeletal system [9, 11–15]. For instance, the prone position is favoured for posterior spinal approaches due to the enhanced access it provides, but requires careful consideration of how it affects internal structures [14, 16–19]. Conversely, the supine position is traditionally used for anterior approaches, offering stability but limited flexibility for posterior manipulations [20–24]. The lateral position, increasingly employed for lateral lumbar interbody fusion (LLIF), oblique anterior lumbar interbody fusion (OLIF), lateral anterior lumbar interbody fusion (LALIF) and single-position lumbar surgeries combining lateral and dorsal procedures simultaneously, presents its challenges, particularly regarding the displacement of major vessels and abdominal organs compared to supine MRI findings [12, 25–40].

Proper surgical positioning is crucial for minimising the risk of complications, optimising surgical access, and ensuring patient safety [5, 11, 41–48]. Different positions alter the body’s anatomy in specific ways, impacting the surgeon’s ability to access target areas, maintain the stability of vital structures, and reduce the risk of inadvertent injury [49]. With the advent of modern surgical techniques, particularly in lateral and anterior lumbar procedures performed in prone or lateral decubitus positions, or combined single-position dorsoventral simultaneous surgeries, these positional considerations become increasingly important. This is especially true in complex spine surgeries, where precision is essential for achieving optimal outcomes.

This review aims to provide a comprehensive analysis of how prone, supine, and lateral positions impact vascular anatomy, abdominal content displacement, and postural alignment during spine surgery. By synthesising findings from multiple studies and conducting a detailed meta-analysis, we aim to provide evidence-based recommendations that inform clinical practice and enhance patient outcomes.

## Methods

### Literature search strategy

**Fig. 1:**
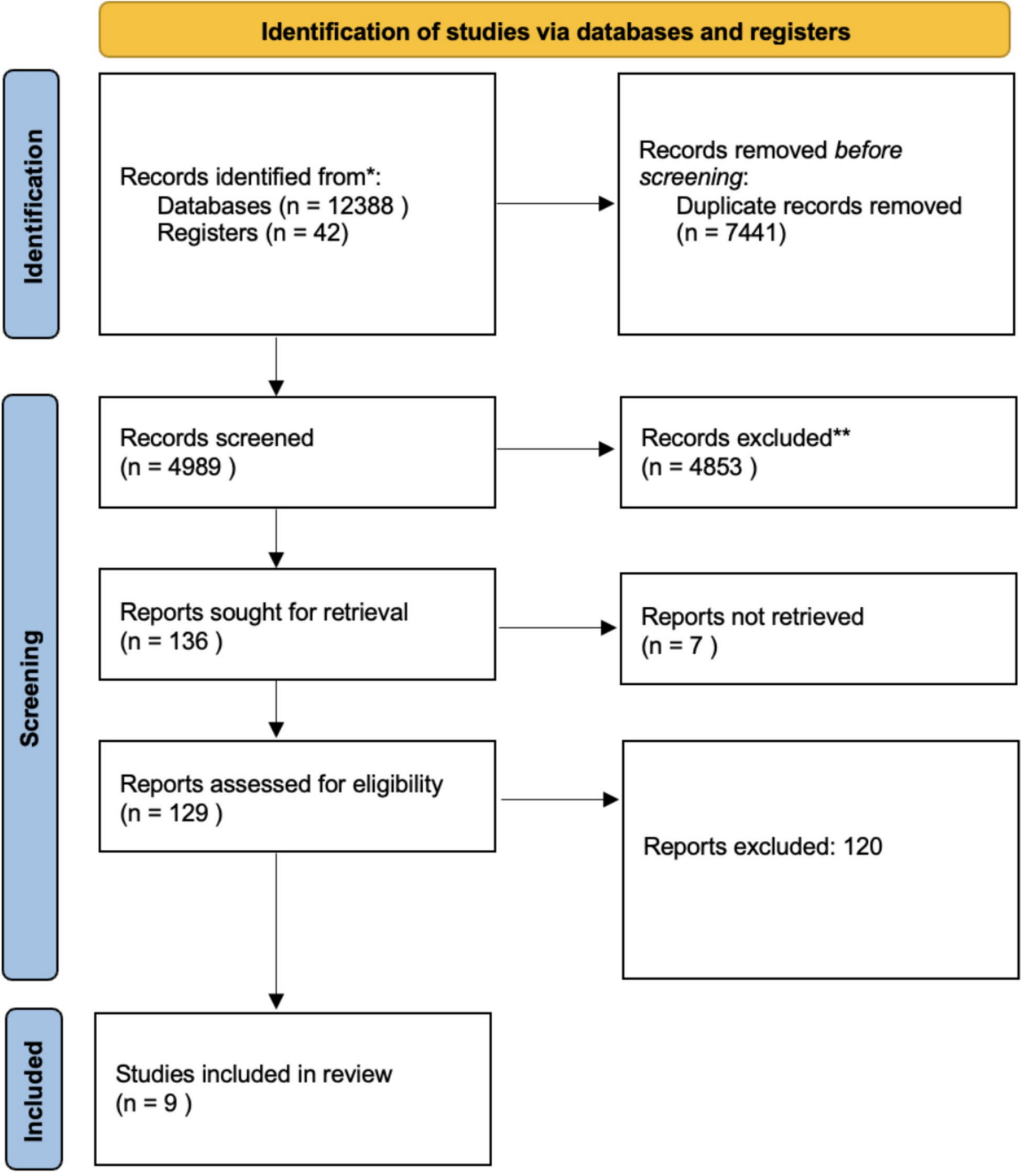
PRISMA 2020 flow diagram for the literature search and study selection

We conducted an updated literature search across multiple databases covering 10 years (January 2015 through March 2025) to ensure all relevant studies were captured. The search included PubMed, MEDLINE (via Ovid), and the Cochrane Library for studies on patient positioning in spine surgery. We combined keywords and medical subject headings related to spinal surgery and patient positioning (e.g., “spine surgery”, “lumbar fusion”, “prone position”, “lateral decubitus”, “supine position”). We applied appropriate Boolean operators to broaden the query. The search was limited to human studies published in the English language. This comprehensive strategy was conducted in accordance with the Preferred Reporting Items for Systematic Reviews and Meta-Analyses (PRISMA) 2020 guidelines, and duplicate records were removed before screening (Fig. 1).

### Screening

Following the removal of duplicates, 3801 records remained. These records were screened based on titles and abstracts. Studies that did not specifically address the impact of surgical positioning on the anatomical or clinical outcomes of interest were excluded. After this phase, 382 studies were selected for full-text review.

### Eligibility

We included original quantitative studies—randomised, quasi‑experimental, or observational—that (1) compared at least two of the three index positions (prone, supine, lateral) in adult spine surgery and (2) reported numeric data on vascular displacement, retroperitoneal organ shift, psoas/nerve plexus position, or segmental lordosis measured in millimetres or degrees. Case reports, cadaveric studies, conference abstracts, and reviews were excluded from the analysis.

After automatic duplicate removal, 290 records remained for title/abstract screening in Rayyan. Two reviewers (A.D. and S.S.) independently screened all titles and abstracts/abstracts and subsequently the full texts of 50 articles; Cohen’s *κ* for inclusion agreement was 0.88. Discrepancies were resolved by consensus or third-reviewer arbitration (F.M.).

### Data extraction

A piloted data‑extraction form (Microsoft Excel) captured:

- Bibliographic details (first author, year, country)
- Study design and sample size
- Index and reference positions (e.g., “supine → prone”)
- Spinal level(s), structure examined (aorta, IVC, colon, psoas, lordosis)
- Imaging modality (CT, MRI, fluoroscopy, ultrasound, intra‑operative radiograph)
- Means, standard deviations (SD), and *n* for each position.

All extractions were performed in duplicate (A.D., S.S.); conflicts were reconciled by discussion. Where data were reported graphically, means and SD were digitised using WebPlotDigitizer 4.6. To ensure consistency across the extracted data, the authors utilised a standardised data extraction form. This form was designed to capture all relevant details consistently across different studies, ensuring that no critical information was overlooked. The form included predefined fields for each variable, and each author independently filled out the form based on their review of the study data. After the initial extraction, the two sets of data were compared, and any discrepancies were resolved through discussion and consensus. This rigorous approach minimised the risk of errors and ensured that the extracted data were reliable and comprehensive.

Studies that lacked quantitative outcomes, did not specify the surgical position, or were reviews/commentaries were excluded. Following this evaluation, 240 studies were excluded due to reasons such as the lack of relevant data or failure to meet the eligibility criteria.

### Inclusion

A total of nine studies were included in the meta-analysis. These studies provided data on the impact of prone, supine, and lateral positions on vascular displacement, abdominal content migration, and changes in lumbar lordosis.

### Risk‑of‑bias assessment

Because all included studies were non‑randomised, ROBINS‑I was applied to seven bias domains (confounding, selection, classification, deviation, missing data, measurement, reporting). Each study was independently rated as low, moderate, serious, or critical risk by two reviewers; M.K. adjudicated disagreements. The risk profile informed sensitivity analyses and the narrative interpretation.

### Statistical methods

The analyses described in this study were performed using the R programming language. The meta and metafor packages were used to conduct the traditional meta-analysis, including the leave-one-out sensitivity analysis. For the Bayesian meta-analysis, the rstanarm package was utilised. This package enables Bayesian modelling using the Stan probabilistic programming language, which is integrated within R. The rstanarm package simplifies the process of fitting Bayesian models, allowing researchers to specify models using familiar R syntax while leveraging the power of Stan for MCMC sampling.

To synthesise all direct and indirect evidence across the three index positions, we ran a frequentist graph-theoretical NMA with the netmeta R package (v 1.5– 2). Treatment effects were expressed as mean differences (MD) and estimated under a common-effects model and a random-effects model using the restricted maximum-likelihood (REML)estimator for τ2τ2. Global heterogeneity was quantified using QQ and I2I2; incoherence between multi-arm designs and the network as a whole was assessed with the design-by-treatment interaction test.

A leave-one-out sensitivity analysis was performed to assess the robustness of the pooled effect sizes. Forest plots were generated to visualise the effect sizes with and without each study, providing clear insights into the stability of the findings. The *I*^2^ statistic was recalculated for each iteration to monitor changes in heterogeneity. The Bayesian meta-analysis was conducted using the Markov Chain Monte Carlo (MCMC) methods to estimate the posterior distributions of the mean effect size and residual standard deviation. Four independent Markov chains of 2000 iterations each (500-iterations warm-up, 1500 samples retained; total posterior draws = 6000) were run in *rstanarm 2.21*. Convergence was confirmed for all monitored parameters (R-hat ≤ 1.01; adequate sample size > 1000). A weakly informative prior Normal(0, 100) on the pooled mean and a *half-Cauchy*(0,10) on *τ* were chosen to let the data dominate while avoiding improper posteriors. The 95% credible intervals provided a clear indication of the uncertainty surrounding the estimates, and the posterior distributions were examined to assess the likelihood of various effect sizes.

## Results

### Study selection and characteristics

The search yielded 12,388 records, of which 4989 titles and abstracts were screened after duplicate removal, 129 full texts were examined, and nine studies containing 41 independent comparisons met all eligibility criteria (PRISMA flow diagram, Fig. 1). Half of the outcome assessments were performed using MRI (49%), the remainder employed CT (34%), ultrasound (10%), or intraoperative fluoroscopy (7%). Vascular displacement accounted for 18 comparisons, retroperitoneal‑organ shift for 7, psoas/plexus position for 6, and segmental lordosis for 10. Detailed study‑level information, including spinal level, structure examined and imaging modality, is provided in Supplement 1.

### Risk of bias and sensitivity analysis

Among the nine contributing studies, two were judged at low overall risk of bias, three at moderate, three at serious and one at critical risk (Table 1). Most concerns arose from confounding by patient selection and unblinded outcome measurement; nevertheless, exclusion of serious/critical studies did not materially change the pooled estimate.

**Table 1:**
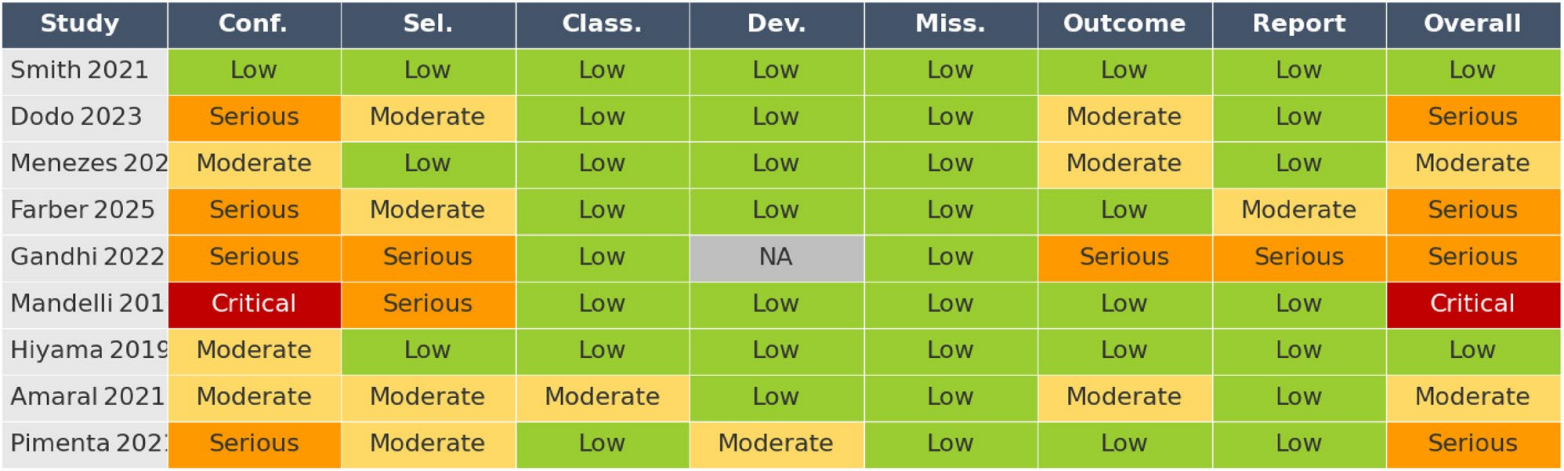
ROBINS-I traffic-light matrix showing domain-level and overall risk-of-bias judgements for the nine included studies. Colours: green = low risk, yellow = moderate, orange = serious, red = critical, grey = not applicable

**Fig. 2:**
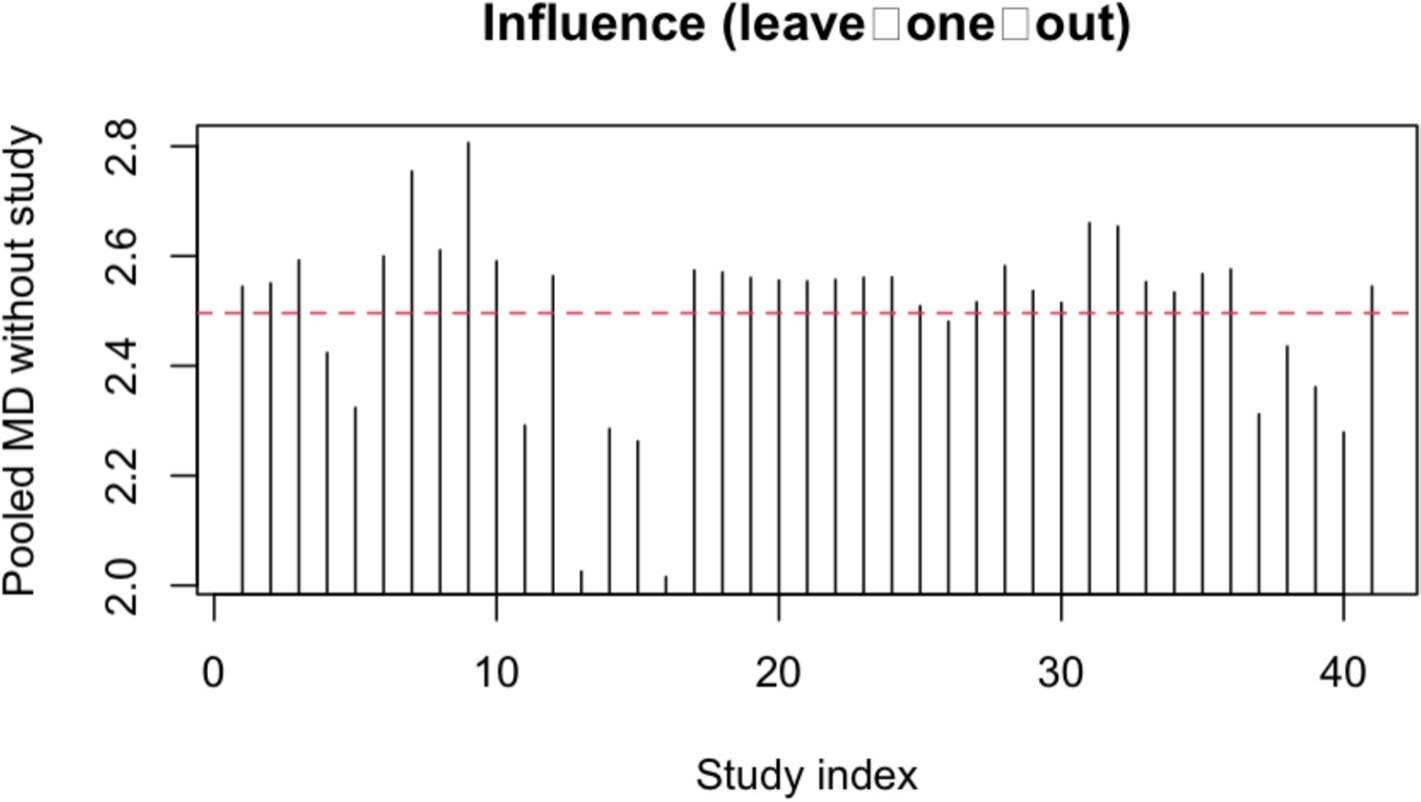
Leave-one-out influence analysis of the pooled mean difference (MD). Each vertical bar shows the pooled MD after omitting one comparison; the dashed red line marks the overall random-effects estimate (+ 2.50 mm/°). None of the 41 deletions moved the pooled effect outside the original 95% CI, confirming the robustness of the result

The leave-one-out influence analysis showed that the pooled MD varied only from + 2.02 mm/° to + 2.81 mm/° across 41 re-fits—well within the original 95% confidence limits. Corresponding *τ*^2^ estimates ranged from 25.2 mm^2^ to 39.4 mm^2^ (Fig. 2).

Re-estimating between-study variance with DerSimonian–Laird or Paule–Mandel methods, and re-running the Bayesian model with a half-Cauchy (0, 10) prior, changed the pooled MD by < 0.4 mm/° and did not affect statistical significance.

### Overall pooled effect and heterogeneity analysis

Across all anatomical structures and position pairs, the random‑effects meta‑analysis produced a mean difference (MD) of + 2.50 mm or degrees (95% CI 0.20–4.79; *p* = 0.034). Between‑study heterogeneity was very high (τ^2^ = 37.7; *I*^2^ = 99.97%), and the 95% prediction interval ranged from –9.7 to + 14.7 mm/°.

### Meta‑regression

A mixed-effects model that included comparison position, imaging modality, and anatomical domain explained only 2.6% of the between-study heterogeneity (*R*^2^ = 2.56%). The omnibus moderator test was non-significant (QM(6) = 5.51; *p* = 0.48; QE(34) = 251.47; *p* < 0.001), and none of the six individual coefficients reached statistical significance (see Supplementary Table S4 for full coefficients and 95% CIs).

### Bayesian analysis

The Bayesian -normal random-effects model converged (all R-hat ≤ 1.01); posterior mean MD =  + 2.8 mm/°, 95% CrI −2.7 to + 8.4 with a posterior between-study SD of τ = 5.9 mm/° (95% CrI 2.3–12.4). Model fit and predictive performance were adequate (elpd < sub > loo < /sub >  = –222.9 ± 41.7;*p* < sub > loo < /sub >  = 30.4 ± 6.7; LOOIC = 445.7 ± 83.5). All 41 Pareto-k diagnostics were < 0.7, indicating reliable leave-one-out importance weights.

### Results syntheses

**Fig. 3:**
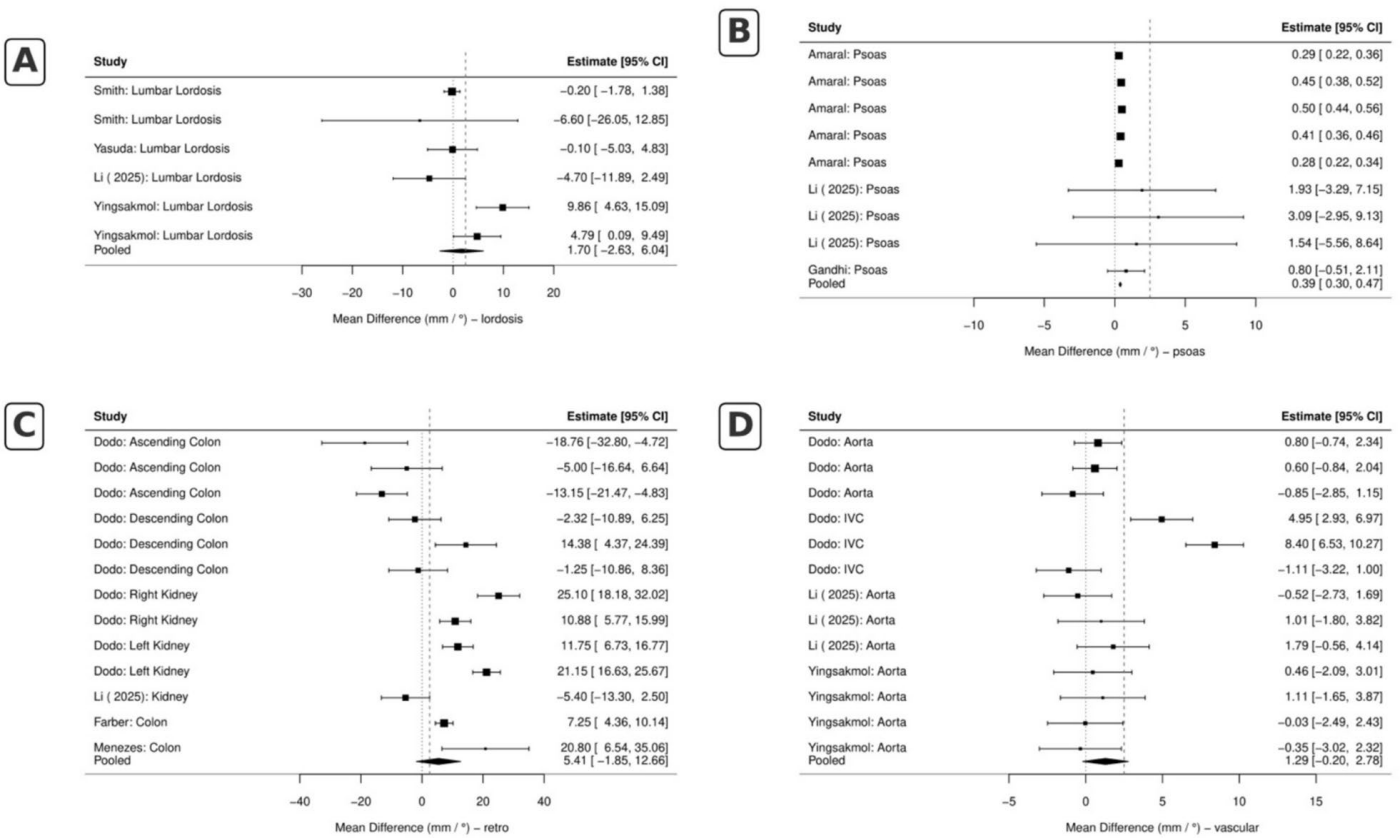
Domain-specific random-effects meta-analyses. A Vascular displacement (18 comparisons); B retroperitoneal-organ shift (7 comparisons); C psoas or lumbar plexus position (6 comparisons); D segmental lordosis (10 comparisons). Grey squares represent study-level mean differences (MD) with size proportional to inverse variance; horizontal lines denote 95% confidence intervals; diamonds show pooled MD (Hartung–Knapp method). Positive values indicate anterior or lateral displacement (in millimetres) or an increase in lordosis (in degrees)

Grouping the 41 comparisons by anatomical domain revealed that only retroperitoneal organs demonstrated a statistically significant pooled displacement (MD + 6.34 mm, 95% CI 1.87–10.80; *p* = 0.007) in the lateral decubitus to prone position. Vascular structures (+ 1.26 mm, 95% CI −2.43–4.94; *p* = 0.49), psoas/plexus position (+ 0.94 mm, 95% CI –3.58–5.46; *p* = 0.68) and segmental lordosis (+ 1.55°, 95% CI −4.62–7.73°; *p* = 0.61) were not different from zero. Full statistics, including heterogeneity, appear in Table 2, and the domain-specific forest plots are displayed in Fig. 3 A–D.

**Table 2:** Domain-specific random-effects meta-analysis. Pooled mean differences (MD) with 95% confidence intervals (CI) are presented for each anatomical domain; *k* = number of independent comparisons; positive MD indicates greater displacement (mm) or lordotic gain (°) in the reference position. *I*^2^ quantifies residual heterogeneity within each subgroup

Domain	*k*	Pooled MD	95% CI	*p*-value	*I*^2^
Retroperitoneal	7	+ 6.34 mm	1.87–10.80	0.007	84%
Vascular structures	18	+ 1.26 mm	− 2.43–4.94	0.49	93%
Psoas/plexus	6	+ 0.94 m	− 3.58–5.46	0.68	88%
Segmental lordosis	10	+ 1.55	− 4.62–7.73	0.61	91%

### Pairwise position contrasts

Mixed‑effects modelling of the three direct position contrasts revealed that the transition from supine to prone produced a significant increase in displacement or lordosis (MD + 3.64 mm/°, 95% CI  0.53–6.76; *p* = 0.023). Neither supine → lateral nor prone → lateral contrasts reached significance.

### Network meta-analysis

In the random-effects model, mean differences (MD) relative to the lateral position were prone –0.16 mm/° (95% CI –3.38 to + 3.06; *p* = 0.92) and supine + 3.01 mm/° (95% CI –0.43 to + 6.44; *p* = 0.086). The indirect contrast between prone and supine was –3.17 mm/° (95% CI –6.54 to + 0.20). Global heterogeneity remained high (*τ*^2^ = 37.50; *I*^2^ = 89.1%), and significant inconsistency was detected (Q < sub > between < /sub >  = 24.6, df = 1, *p* < 0.0001).

## Discussion

This meta-analysis of 9 studies (41 comparisons) demonstrates that patient positioning alters spinal surgical anatomy selectively rather than uniformly. Two findings are consistent and clinically actionable: retroperitoneal organs shift posteriorly by 6 mm from the lateral to the prone position, and moving from supine to prone increases combined displacement/lordosis by 3–4 mm/°.

All other pooled effects—vascular, psoas/plexus, segmental lordosis—were small and highly variable, as reflected by an overall prediction interval of –9.7 to + 14.7 mm/°.

Robustness and uncertainty were explored systematically. Leave-one-out influence analysis confirmed that no single comparison shifted the pooled estimate outside its original 95% CI, indicating that any outlier study did not drive the findings. Meta-regression incorporating position pair, imaging modality, and anatomical domain explained only 2.6% of between-study variance, and none of the individual moderators reached significance. Hence, the high heterogeneity (*I*^2^ ≈ 100%) remains unexplained mainly at the study level. Risk-of-bias stratification showed one-third of studies at serious/critical risk, yet omitting them changed the pooled MD by < 0.4 mm/° and left directionality unchanged, supporting the stability of the central conclusions despite methodological limitations.

Mean vessel displacement did not differ statistically among prone, supine and lateral positions. A thorough understanding of the vascular anatomy is imperative before surgery, as the risk is particularly heightened for less experienced surgeons, who may be more likely to inadvertently injure the inferior vena cava (IVC), given that individual patients still exhibit vessel migrations of up to one centimetre. Although the absolute excursions are small, even a 3–5 mm medial shift of the IVC can propagate distally along the common trunk and produce a comparable medialisation of the internal iliac veins at L5–S1, potentially narrowing the anterior corridor for cage insertion or screw placement at the lumbosacral junction. Gandhi et al. showed that posture changes can shift the aorta and inferior vena cava by only a few millimetres in either approach: in the lateral decubitus (with hip flexion), the aorta moved slightly lateral toward the approach side, while in prone, the aorta remained more central and the IVC shifted slightly medial [50]. Importantly, these authors noted that the calibre of the IVC changed with positioning—it appeared more engorged (“full and open”) in the lateral decubitus orientation. It became flattened in supine or prone positions due to compression by abdominal contents. A compressed vein may present a smaller profile, but it does not vanish from the operative field, and the aorta’s position remains relatively fixed by its tethering [49]. No clinical study to date has demonstrated a significantly lower incidence of vascular injury with prone LLIF versus traditional lateral LLIF, which aligns with the anatomical observations above. Notably, large surveys of standard lateral transpsoas fusion have documented extremely low rates of catastrophic vascular or visceral injuries—on the order of 0.1% or less for major vessel laceration and ~ 0.08% for bowel injury [27, 51]. The use of patient-specific preoperative planning, including 3D modelling or CT scans in the lateral decubitus position, may help to anticipate potential complications and tailor the surgical approach to the individual patient’s anatomy [10, 52–56]. An intraoperative CT in the lateral decubitus position could provide a clearer understanding of these anatomical shifts [35, 57, 58]. Combined with navigated instrumentation, this method can address these anatomical changes, significantly reducing the risk of nerve and venous injuries while also shortening operative time (through a simultaneous dorsal approach) and enhancing overall surgical safety and efficacy [32, 59, 60]. Additionally, the use of neuromonitoring can provide valuable feedback on the status of neural structures, helping to prevent nerve damage and other neurological complications [61–66].

This study reveals a 6 mm posterior migration of the colon and kidney, which narrows the surgical corridor during anterior or anterolateral approaches at L2–L4. Nevertheless, clinically minimal displacement of abdominal contents in the supine position reaffirms its status as the preferred position for anterior spinal procedures. This interpretation is consistent with recent anatomical studies, which show that abdominal contents do not fall away substantially more in prone positioning than in the lateral decubitus orientation [67].

Dodo et al. observed that prone positioning caused a modest ventral shift of specific organs compared to supine imaging. However, a considerable proportion of patients (up to 88.6%) still had retroperitoneal structures within 10 mm of the disc space even when [68]. Similarly, Farber et al. reported that the overall magnitude of bowel migration was small and not significantly different between prone-extension and lateral decubitus positions [67]. Conventional lateral decubitus positioning with table flexion is already known to shift abdominal organs anteriorly and enlarge the retroperitoneal working space [69]. In a prospective CT study, Ouchida et al. demonstrated that lateral positioning significantly decreased the presence of organs within the operative “approach zone” compared to supine imaging [69]. However, even with the patient in the lateral decubitus position, 83% of upper lumbar levels still had the kidney overlapping the surgical corridor, and 20% had the descending colon encroaching on the disk space [69]. Menezes et al. measured a pronounced posterior migration of the intra-abdominal contents at the L4–L5 level in prone patients, which effectively halved the safe distance between the colon and the disc [70]. All subjects in their MRI series exhibited a posterior colon shift when placed in the prone position, indicating a uniform trend that potentially narrows the working window at L4–L5 [70]. These results raise concern that, without meticulous retraction and localisation, the risk of bowel contact or injury may be higher at the L4–L5 level during prone lateral fusion than in the standard lateral position. However, the supine position also presents certain limitations, particularly in terms of accessing the retroperitoneal space. The abdominal contents, including the intestines and peritoneum, lie directly over the operative site, complicating their mobilisation and increasing the risk of complications such as bowel injury or postoperative ileus if not handled meticulously. Although the supine position is well-suited for anterior lumbar interbody fusion (ALIF), it may necessitate adjunctive techniques to achieve optimal surgical outcomes when posterior access is required, demanding careful planning and execution to balance the anatomical constraints [12, 27, 71].

Prone positioning remains advantageous for sagittal realignment, as shown in this study; the statistically significant 3–4° lordosis gain can reduce the need for aggressive osteotomies. This postural advantage was anticipated: placing the patient prone naturally increases lumbar lordosis by allowing the abdomen to sag and the lumbar spine to extend. Smith et al. quantified this effect in healthy adults and found that all prone configurations yielded greater lumbar lordosis than the lateral decubitus position on a standard table [72]. In their fluoroscopic analysis, the lateral decubitus position (even with table break) produced the least lordosis. In contrast, prone positioning, especially with hips extended and gentle downward pressure or table extension, is associated with the greatest lordosis [72]. This innate increase in lordotic alignment translates into real surgical benefits when inserting interbody cages. Pimenta et al. reported that the prone transpsoas technique achieved a mean increase of ~ 6° in segmental lordosis at the indexed levels, significantly higher than the preoperative baseline and resulting in improved global alignment [73]. By comparison, traditional lateral decubitus LLIF typically yields about 3–4° of segmental lordotic gain per level with lordotic cages [73]. Thus, prone positioning roughly doubles the segmental correction potential relative to the same procedure performed in lateral decubitus. In Pimenta’s series, this translated to better overall sagittal profiles: the proportion of patients with a high pelvic incidence–lumbar lordosis mismatch (> 10°) was reduced from 22% preoperatively to 12% postoperatively with prone LLIF, representing a significant improvement in spinopelvic alignment [73]. These outcomes are comparable to, or even exceed, the lordotic corrections reported for anterior lumbar interbody fusion (ALIF) in some studies. Ahlquist et al. found that lateral interbody fusion and ALIF both produce superior radiographic lordosis gains compared to posterior fusions, with single-level LLIF improving the segmental angle by ~ 4.4° on average and ALIF by ~ 7.9° [74]. The prone lateral approach appears to bridge some of this gap by allowing a lateral cage to achieve a lordotic correction closer to that of ALIF, likely because the disc space is oriented in a more extended posture during cage insertion. Additionally, prone positioning may facilitate better access and cage placement at the L4–L5 level. Prior analyses have noted that the iliac crest can impede the trajectory for L4–L5 in lateral decubitus, sometimes necessitating table flexion or partial osteotomy of the crest [72]. In the prone position, however, the use of an adjustable frame that permits slight coronal bending of the patient can improve the L4–L5 accessibility around the iliac crest, while simultaneously capitalising on gravity to increase lordosis [72]. This dual benefit is unique to the prone lateral technique. It may be especially advantageous in patients requiring maximal lordotic restoration at L4–L5 as part of a deformity correction or to achieve global alignment.

The findings of this meta-analysis have significant implications for current surgical practices. Surgeons must account for the anatomical shifts associated with each patient position and select the most suitable strategy based on the surgery’s objectives and the patient’s specific anatomy. For instance, in procedures where spinal alignment is a primary goal, the prone position’s ability to enhance lordosis provides a critical advantage. Combining anterior and posterior access simultaneously, while leveraging gravity, makes this approach particularly appealing for complex realignment procedures such as Schwab osteotomies, as it eliminates the need for repositioning during surgery. It is essential to recognise that the anatomy of the psoas muscle in the prone position is likely similar to that in the supine position.

Additionally, when the abdomen is allowed to hang freely due to gravity, using proper positioning aids such as bolsters or specialised surgical tables, the abdominal contents naturally shift downward, creating a comparable retroperitoneal space for retractor placement. Future MRI studies in the prone position, with the abdomen hanging and the chest and pelvis supported on bolsters, could help validate this hypothesis by providing clearer insights into these anatomical dynamics. However, this approach is limited by anatomical structures such as the ribs and iliac crest, which can complicate access to levels like L4/5 or above L2/3.

Conversely, for surgeries requiring stability and minimal interference with abdominal contents, the supine position offers several advantages. It allows for optimal visualisation of the surgical field, including vascular, intra-abdominal, and retroperitoneal structures, making it ideal for managing complications and ensuring precise control throughout the procedure. A significant drawback lies in the influence of gravity, as abdominal structures are forced against the retractors, requiring extended effort to counteract this pressure. Furthermore, the inability to perform a simultaneous posterior approach introduces constraints, particularly in addressing severe spinal misalignments, limiting the surgeon’s capacity for comprehensive correction.

Strengths of the study include the largest dataset to date, risk-of-bias stratification, and concordant findings across frequentist, network and Bayesian models. Limitations are persistent heterogeneity and an observational study design. While the meta-analysis focused on immediate anatomical changes during surgery, it does not provide information on the long-term impact of these positional changes on patient outcomes, such as recovery time, pain levels, and the need for revision surgery [18]. Future studies should address this gap by including long-term follow-up data to assess the durability of the surgical outcomes associated with different positions.

Prospective imaging studies that scan patients in all three positions, correlation of anatomical shifts with operative metrics and outcomes, and development of torque-responsive operating tables and soft-tissue-aware navigation are priority areas.

## Conclusion

This meta-analysis highlights the critical impact of surgical positioning on outcomes in spine surgery. The prone position enhances spinal realignment and improves lateral retractor placement. In contrast, the supine position offers stability and alignment with standard imaging techniques, albeit with limitations on combined anterior–posterior approaches. The lateral position remains ideal for specific procedures but requires careful vascular management. Advancing imaging and positioning techniques will be key to further optimising surgical outcomes.

## Supplementary Information

Supplementary material 1.

## Data Availability

No datasets were generated or analysed during the current study.

## References

[1] Pascucci S, Langella F, Franzo M, Tesse MG, Ciminello E, Biondi A, et al. National spine surgery registries’ characteristics and aims: globally accepted standards have yet to be met. Results of a scoping review and a complementary survey. J Orthop Traumatol. 2023;24(1):49. 10.1186/s10195-023-00732-4. 37715871 10.1186/s10195-023-00732-4 PMC10505129

[2] Luengo-Matos S, Sanchez-Gomez LM, Hijas-Gomez AI, Garcia-Carpintero EE, Ballesteros-Masso R, Polo-deSantos M. Efficacy and safety of robotic spine surgery: systematic review and meta-analysis. J Orthop Traumatol. 2022;23(1):49. 10.1186/s10195-022-00669-0. 36242652 10.1186/s10195-022-00669-0 PMC9569281

[3] Di Martino A, Papalia R, Caldaria A, Torre G, Denaro L, Denaro V. Should evoked potential monitoring be used in degenerative cervical spine surgery? A systematic review. J Orthop Traumatol. 2019;20(1):19. 10.1186/s10195-019-0524-4. 30941518 10.1186/s10195-019-0524-4 PMC6445897

[4] Randelli F, Romanini E, Biggi F, Danelli G, Della Rocca G, Laurora NR, et al. II Italian intersociety consensus statement on antithrombotic prophylaxis in orthopaedics and traumatology: arthroscopy, traumatology, leg immobilization, minor orthopaedic procedures and spine surgery. J Orthop Traumatol. 2013;14(1):1–13. 10.1007/s10195-012-0214-y. 23224149 10.1007/s10195-012-0214-y PMC3585990

[5] Scrofani R, Migliorini F, Smajic S, De Simone M, Maffulli N, Iaconetta G. Percutaneous transforaminal endoscopic discectomy in patients with lumbar disc herniation: a meta-analysis. Eur J Orthop Surg Traumatol. 2025;35(1):276. 10.1007/s00590-025-04374-6. 40553167 10.1007/s00590-025-04374-6

[6] Goyal R, Hooda B, Singh S, Taank P, Mishra A, Singh A. Effects of prone positioning on cerebral oxygenation in patients undergoing spine surgery under general anaesthesia. J Perioper Pract. 2025. 10.1177/17504589251329242. 40152230 10.1177/17504589251329242

[7] Yamaguchi K, Morimoto T, Hirata H, Mawatari M. Letter to the editor: patient positioning in spine surgery: what spine surgeons should know? Asian Spine J. 2023;17(6):1176–7. 10.31616/asj.2023.0353.r1. 38171027 10.31616/asj.2023.0353.r1 PMC10764130

[8] Garg B, Bansal T, Mehta N, Sharan AD. Patient positioning in spine surgery: what spine surgeons should know? Asian Spine J. 2023;17(4):770–81. 10.31616/asj.2022.0320. 37226380 10.31616/asj.2022.0320 PMC10460667

[9] Cunha PD, Barbosa TP, Correia G, Silva R, Cruz Oliveira N, Varanda P, et al. The ideal patient positioning in spine surgery: a preventive strategy. EFORT Open Rev. 2023;8(2):63–72. 10.1530/EOR-22-0135. 36805330 10.1530/EOR-22-0135 PMC9968999

[10] Fan S, Luo H, Chen S, Xiang H, Mai Q, Zhu Z, et al. Effect of different lumbar-iliac fixation and sacral slope for Tile C1.3 pelvic fractures: a biomechanical study. J Orthop Traumatol. 2024;25(1):32. 10.1186/s10195-024-00776-0. 38926180 10.1186/s10195-024-00776-0 PMC11208344

[11] Pappalardo G, Pola E, Bertini FA, Nasto LA, Eschweiler J, Schafer L, et al. Superior mesenteric artery syndrome following spine surgery in idiopathic adolescent scoliosis: a systematic review. Eur J Med Res. 2024;29(1):410. 10.1186/s40001-024-02002-3. 39118170 10.1186/s40001-024-02002-3 PMC11308422

[12] Migliorini F, de Maria N, Tafuri A, Porcaro AB, Rubilotta E, Balzarro M, et al. Late diagnosis of ureteral injury from anterior lumbar spine interbody fusion surgery: case report and literature review. Urologia. 2023;90(3):579–83. 10.1177/03915603211030230. 34251292 10.1177/03915603211030230

[13] Bansal T, Garg B. Response to letter to the editor: patient positioning in spine surgery: what spine surgeons should know? Asian Spine J. 2023;17(6):1178–9. 10.31616/asj.2023.0353.r2. 38171028 10.31616/asj.2023.0353.r2 PMC10764136

[14] Musti S, Chakrabarti D, Bansal S. The effect of prone positioning on surgical pleth index in patients undergoing spine surgery under general anesthesia - a prospective observational study. J Anaesthesiol Clin Pharmacol. 2022;38(4):646–51. 10.4103/joacp.JOACP_39_21. 36778835 10.4103/joacp.JOACP_39_21 PMC9912877

[15] Kolb B, Large J, Watson S, Smurthwaite G. An innovative prone positioning system for advanced deformity and frailty in complex spine surgery. J Neurosurg Spine. 2020;32(2):229–34. 10.3171/2019.7.SPINE19161. 31653810 10.3171/2019.7.SPINE19161

[16] Yang XY, Wei MM, Tan H, Wang HL, Luo MQ, Xu M, et al. The effect of restrictive vs. liberal fluid protocols on ocular parameters in patients undergoing prone spine surgery: a randomized controlled trial. Perioper Med. 2023;12(1):23. 10.1186/s13741-023-00310-6. 10.1186/s13741-023-00310-6 PMC10262396 37308905

[17] Oh SK, Lim BG, Kim H, Lee JH, Lee JE. Performance of a new auxiliary device based on wrist brace to improve accuracy and feasibility in neuromuscular monitoring with acceleromyography in prone-positioned patients undergoing lumbar spine surgery: a prospective randomized clinical trial. J Clin Monit Comput. 2023;37(4):993–1001. 10.1007/s10877-023-01000-w. 37004664 10.1007/s10877-023-01000-w

[18] Kukralova L, Dostalova V, Cihlo M, Kraus J, Dostal P. The impact of individualized hemodynamic management on intraoperative fluid balance and hemodynamic interventions during spine surgery in the prone position: a prospective randomized trial. Medicina. 2022. 10.3390/medicina58111683. 36422222 10.3390/medicina58111683 PMC9698539

[19] Khouri DB, Delgado MA, Lemes JL, Afonso Cruz MM. Differential diagnosis of intraoperative cardiac arrest after spine surgery in prone position. Saudi J Anaesth. 2022;16(4):485–7. 10.4103/sja.sja_893_21. 36337392 10.4103/sja.sja_893_21 PMC9630707

[20] Barber LA, Lafage R, Muzammil H, Shinn DJ, Kim JH, Lafage V, et al. Supine and dynamic extension radiographs as the strongest predictors of post-operative alignment in minimally invasive lumbar spine surgery. Glob Spine J. 2023;13(8):2278–84. 10.1177/21925682221079601. 10.1177/21925682221079601 PMC10538306 35192407

[21] Yoshida G, Ushirozako H, Imagama S, Kobayashi K, Ando K, Ando M, et al. Transcranial motor-evoked potential alert after supine-to-prone position change during thoracic ossification in posterior longitudinal ligament surgery: a prospective multicenter study of the monitoring committee of the Japanese Society for Spine Surgery and Related Research. Spine. 2022;47(14):1018–26. 10.1097/BRS.0000000000004246. 34610608 10.1097/BRS.0000000000004246

[22] Mulukutla RD, Yelamarthy PKK, Vadapalli R. Cortical blindness after cervical spine surgery in supine position - a rare case report and review of the literature. Asian J Neurosurg. 2021;16(2):406–11. 10.4103/ajns.AJNS_473_20. 34268176 10.4103/ajns.AJNS_473_20 PMC8244719

[23] Yoon HK, Lee HC, Chung J, Park HP. Predictive factors for hypotension associated with supine-to-prone positional change in patients undergoing spine surgery. J Neurosurg Anesthesiol. 2020;32(2):140–6. 10.1097/ANA.0000000000000565. 30475290 10.1097/ANA.0000000000000565

[24] Zhou J, Hu A, Zhou X, Dong J. Anterior cervical discectomy and fusion with self-locking standalone cage for the treatment of cervical degenerative disc disease in patients over 80 years. J Orthop Traumatol. 2025;26(1):7. 10.1186/s10195-025-00820-7. 39881082 10.1186/s10195-025-00820-7 PMC11780053

[25] Ozgur BM, Aryan HE, Pimenta L, Taylor WR. Extreme lateral interbody fusion (XLIF): a novel surgical technique for anterior lumbar interbody fusion. Spine J. 2006;6(4):435–43. 10.1016/j.spinee.2005.08.012. 16825052 10.1016/j.spinee.2005.08.012

[26] Tatsumi R, Lee YP, Khajavi K, Taylor W, Chen F, Bae H. In vitro comparison of endplate preparation between four mini-open interbody fusion approaches. Eur Spine J. 2015;24(Suppl 3):372–7. 10.1007/s00586-014-3708-x. 25874742 10.1007/s00586-014-3708-x

[27] Quack V, Eschweiler J, Prechtel C, Migliorini F, Betsch M, Maffulli N, et al. L4/5 accessibility for extreme lateral interbody fusion (XLIF): a radiological study. J Orthop Surg Res. 2022;17(1):483. 10.1186/s13018-022-03320-0. 36369101 10.1186/s13018-022-03320-0 PMC9652979

[28] Baroncini A, Berjano P, Migliorini F, Lamartina C, Vanni D, Boriani S. Rapidly destructive osteoarthritis of the spine: lessons learned from the first reported case. BMC Musculoskelet Disord. 2022;23(1):735. 10.1186/s12891-022-05686-y. 35915481 10.1186/s12891-022-05686-y PMC9340694

[29] El Mansy Y, Migliorini F, Tingart M, Madarassy G. Minimally versus conventional-invasive transforaminal lumbar interbody fusion in patients with failed back surgery syndrome. Musculoskelet Surg. 2021;105(3):297–302. 10.1007/s12306-020-00659-7. 32319074 10.1007/s12306-020-00659-7

[30] Ferraro M, Puglia F, Della Valle A, Cerbone V, Cicatelli A, Peroni DR, et al. Transforaminal lumbar interbody fusion with a tantalum cage: lumbar lordosis redistribution and sacral slope restoration with a modified posterior technique. J Orthop Traumatol. 2023;24(1):62. 10.1186/s10195-023-00741-3. 38091159 10.1186/s10195-023-00741-3 PMC10719190

[31] Ukai T, Katoh H, Yokoyama K, Sato M, Watanabe M. Effect of spinal fusion on joint space narrowing of the hip: comparison among non-fusion, short fusion, and middle or long fusion. J Orthop Traumatol. 2023;24(1):1. 10.1186/s10195-022-00682-3. 36622495 10.1186/s10195-022-00682-3 PMC9829947

[32] La Rocca G, Mazzucchi E, Pignotti F, Nasto LA, Galieri G, Olivi A, et al. Intraoperative CT-guided navigation versus fluoroscopy for percutaneous pedicle screw placement in 192 patients: a comparative analysis. J Orthop Traumatol. 2022;23(1):44. 10.1186/s10195-022-00661-8. 36048284 10.1186/s10195-022-00661-8 PMC9437178

[33] Kumar BS, Tanaka M, Arataki S, Fujiwara Y, Mushtaq M, Taoka T, et al. Lateral access minimally invasive spine surgery in adult spinal deformity. J Orthop. 2023;45:26–32. 10.1016/j.jor.2023.09.007. 37822643 10.1016/j.jor.2023.09.007 PMC10562616

[34] Kim SH, Kim JH, Kwon JW, Kim HS, Moon SH, Suk KS, et al. Assessment of biomechanical advantages in combined anterior-posterior cervical spine surgery by radiological outcomes: pedicle screws over lateral mass screws. J Clin Med. 2023. 10.3390/jcm12093201. 37176646 10.3390/jcm12093201 PMC10179026

[35] Hiyama A, Sakai D, Katoh H, Nomura S, Watanabe M. Assessing procedural accuracy in lateral spine surgery: a retrospective analysis of percutaneous pedicle screw placement with intraoperative CT navigation. J Clin Med. 2023. 10.3390/jcm12216914. 37959378 10.3390/jcm12216914 PMC10647313

[36] Henao Romero S, Berbeo M, Diaz R, Villamizar Torres D. Minimally invasive lateral single-position surgery for multilevel degenerative lumbar spine disease: feasibility and perioperative results in a single Latin-American spine center. Eur Spine J. 2023;32(5):1688–94. 10.1007/s00586-023-07591-x. 36961569 10.1007/s00586-023-07591-x

[37] Soliman MAR, Khan A, Pollina J. Comparison of prone transpsoas and standard lateral lumbar interbody fusion surgery for degenerative lumbar spine disease: a retrospective radiographic propensity score-matched analysis. World Neurosurg. 2022;157:e11–21. 10.1016/j.wneu.2021.08.097. 34464774 10.1016/j.wneu.2021.08.097

[38] Li J, Chen Y, Wu H, Gan K, Bei D, Fan T, et al. Can oblique lateral interbody fusion (OLIF) create more lumbosacral lordosis in lumbar spine surgery than minimally invasive transforaminal interbody fusion (MIS-TLIF)? Front Surg. 2022;9:1063354. 10.3389/fsurg.2022.1063354. 36684176 10.3389/fsurg.2022.1063354 PMC9852049

[39] Ishihara M, Taniguchi S, Kawashima K, Adachi T, Paku M, Tani Y, et al. Bone fusion morphology after circumferential minimally invasive spine surgery using lateral lumbar interbody fusion and percutaneous pedicle screws without bone grafting in the thoracic spine: a retrospective study. Medicina. 2022;58(4):496. 10.3390/medicina58040496. 35454335 10.3390/medicina58040496 PMC9031519

[40] Inoue D, Shigematsu H, Matsumori H, Ueda Y, Tanaka Y. Accuracy of lateral mass screw insertion during cervical spine surgery without fluoroscopic guidance and comparison of postoperative screw loosening rate among unicortical and bicortical screws using computed tomography. Spine Surg Relat Res. 2022;6(6):625–30. 10.22603/ssrr.2022-0055. 36561156 10.22603/ssrr.2022-0055 PMC9747216

[41] Migliorini F, Pilone M, Eschweiler J, Katusic D, Memminger MK, Maffulli N. Therapeutic strategies that modulate the acute phase of secondary spinal cord injury scarring and inflammation and improve injury outcomes. Expert Rev Neurother. 2025;25(4):477–90. 10.1080/14737175.2025.2470326. 40042224 10.1080/14737175.2025.2470326

[42] Pishnamaz M, Migliorini F, Blume C, Kobbe P, Trobisch P, Delbruck H, et al. Long-term outcomes of spinal fusion in adolescent idiopathic scoliosis: a literature review. Eur J Med Res. 2024;29(1):534. 10.1186/s40001-024-02052-7. 39497199 10.1186/s40001-024-02052-7 PMC11536752

[43] Pappalardo G, Schneider S, Kotsias A, Jeyaraman M, Schafer L, Migliorini F. Negative pressure wound therapy in the management of postoperative spinal wound infections: a systematic review. Eur J Orthop Surg Traumatol. 2024;34(5):2303–13. 10.1007/s00590-024-03983-x. 38753028 10.1007/s00590-024-03983-x

[44] Migliorini F, Maffulli N, Schäfer L, Manocchio N, Bossa M, Foti C, et al. Impact of education in patients undergoing physiotherapy for lower back pain: a level I systematic review and meta-analysis. Eur J Trauma Emerg Surg. 2025;51(1):113. 10.1007/s00068-025-02788-9. 39969656 10.1007/s00068-025-02788-9 PMC11839871

[45] Michalik R, Kuhlmann B, Wild M, Siebers HL, Migliorini F, Eschweiler J, et al. The effect of breast size on spinal posture. Aesthet Plast Surg. 2024;48(7):1331–8. 10.1007/s00266-022-03141-w. 10.1007/s00266-022-03141-w PMC11035396 36280605

[46] Baroncini A, Maffulli N, Pilone M, Pipino G, Memminger MK, Pappalardo G, et al. Prognostic factors in patients undergoing physiotherapy for chronic low back pain: a level i systematic review. J Clin Med. 2024. 10.3390/jcm13226864. 39598010 10.3390/jcm13226864 PMC11594606

[47] Audisio A, Aprato A, Reinaudo V, Sinatra G, Lucchino L, Masse A. Endoscopic-assisted percutaneous fixation for displaced anterior inferior iliac spine avulsion fractures: a prospective cohort study. J Orthop Traumatol. 2025;26(1):16. 10.1186/s10195-025-00831-4. 40057584 10.1186/s10195-025-00831-4 PMC11890690

[48] Wang M, Wang X, Wang H, Shen Y, Qiu Y, Sun X, et al. Validation of Roussouly classification in predicting the occurrence of adjacent segment disease after short-level lumbar fusion surgery. J Orthop Traumatol. 2024;25(1):2. 10.1186/s10195-023-00744-0. 38217751 10.1186/s10195-023-00744-0 PMC10787724

[49] Pimenta L, Turner AW, Dooley ZA, Parikh RD, Peterson MD. Biomechanics of lateral interbody spacers: going wider for going stiffer. Sci World J. 2012;2012:381814. 10.1100/2012/381814. 10.1100/2012/381814 PMC3504399 23213284

[50] Gandhi SV, Dugan R, Farber SH, Godzik J, Alhilali L, Uribe JS. Anatomical positional changes in the lateral lumbar interbody fusion. Eur Spine J. 2022;31(9):2220–6. 10.1007/s00586-022-07195-x. 35428915 10.1007/s00586-022-07195-x

[51] Uribe JS, Deukmedjian AR. Visceral, vascular, and wound complications following over 13,000 lateral interbody fusions: a survey study and literature review. Eur Spine J. 2015;24(Suppl 3):386–96. 10.1007/s00586-015-3806-4. 25720864 10.1007/s00586-015-3806-4

[52] Chumchuen S, Lertpullpol W, Apivatgaroon A. Open technique for supra-acetabular pin placement in pelvic external fixation: a cadaveric study. J Orthop Traumatol. 2022;23(1):14. 10.1186/s10195-022-00635-w. 35286486 10.1186/s10195-022-00635-w PMC8921377

[53] Okada R, Sakai T, Nishisho T, Nitta A, Takahara S, Oba K, et al. Preoperative planning using three-dimensional printing for full-endoscopic spine surgery: a technical note. NMC Case Rep J. 2022;9:249–53. 10.2176/jns-nmc.2022-0077. 36128054 10.2176/jns-nmc.2022-0077 PMC9458159

[54] Muralidharan V, Swaminathan G, Devadhas D, Joseph BV. Patient-specific interactive software module for virtual preoperative planning and visualization of pedicle screw entry point and trajectories in spine surgery. Neurol India. 2018;66(6):1766–70. 10.4103/0028-3886.246281. 30504578 10.4103/0028-3886.246281

[55] Janjua MB, Tishelman JC, Vasquez-Montes D, Vaynrub M, Errico TJ, Buckland AJ, et al. The value of sitting radiographs: analysis of spine flexibility and its utility in preoperative planning for adult spinal deformity surgery. J Neurosurg Spine. 2018;29(4):414–21. 10.3171/2018.2.SPINE17749. 29979136 10.3171/2018.2.SPINE17749

[56] Piazzolla A, Bizzoca D, Barbanti-Brodano G, Formica M, Pietrogrande L, Tarantino U, et al. Capacitive biophysical stimulation improves the healing of vertebral fragility fractures: a prospective multicentre randomized controlled trial. J Orthop Traumatol. 2024;25(1):17. 10.1186/s10195-024-00758-2. 38622334 10.1186/s10195-024-00758-2 PMC11018575

[57] Eastman JG, Routt ML Jr. Correlating preoperative imaging with intraoperative fluoroscopy in iliosacral screw placement. J Orthop Traumatol. 2015;16(4):309–16. 10.1007/s10195-015-0363-x. 26195031 10.1007/s10195-015-0363-x PMC4633422

[58] Banat M, Wach J, Salemdawod A, Domurath L, Scorzin J, Vatter H. Can postoperative CT imaging in spine surgery be replaced by intraoperative 3D rotation with the C-arm?: results of a prospective single center cohort study. Front Surg. 2021;8:692189. 10.3389/fsurg.2021.692189. 34336918 10.3389/fsurg.2021.692189 PMC8321091

[59] Mandelli C, Colombo EV, Sicuri GM, Mortini P. Lumbar plexus nervous distortion in XLIF(®) approach: an anatomic study. Eur Spine J. 2016;25(12):4155–63. 10.1007/s00586-016-4617-y. 27220971 10.1007/s00586-016-4617-y

[60] La Rocca G, Mazzucchi E, Pignotti F, Nasto LA, Galieri G, Rinaldi P, et al. Navigated, percutaneous, three-step technique for lumbar and sacral screw placement: a novel, minimally invasive, and maximally safe strategy. J Orthop Traumatol. 2023;24(1):32. 10.1186/s10195-023-00696-5. 37386233 10.1186/s10195-023-00696-5 PMC10310656

[61] Hiyama A, Katoh H, Sakai D, Sato M, Tanaka M, Watanabe M. Comparison of radiological changes after single- position versus dual- position for lateral interbody fusion and pedicle screw fixation. BMC Musculoskelet Disord. 2019;20(1):601. 10.1186/s12891-019-2992-3. 31830959 10.1186/s12891-019-2992-3 PMC6909463

[62] Wilson JP, Vallejo JB, Kumbhare D, Guthikonda B, Hoang S. The use of intraoperative neuromonitoring for cervical spine surgery: indications, challenges, and advances. J Clin Med. 2023. 10.3390/jcm12144652. 37510767 10.3390/jcm12144652 PMC10380862

[63] Ament JD, Leon A, Kim KD, Johnson JP, Vokshoor A. Intraoperative neuromonitoring in spine surgery: large database analysis of cost-effectiveness. N Am Spine Soc J. 2023;14:100206. 10.1016/j.xnsj.2023.100206. 37008516 10.1016/j.xnsj.2023.100206 PMC10064224

[64] Daroszewski P, Garasz A, Huber J, Kaczmarek K, Janusz P, Glowka P, et al. Update on neuromonitoring procedures applied during surgery of the spine - observational study. Reumatologia. 2023;61(1):21–9. 10.5114/reum/160209. 36998584 10.5114/reum/160209 PMC10044034

[65] Chandra AA, Vaishnav A, Shahi P, Song J, Mok J, Alluri RK, et al. The role of intraoperative neuromonitoring modalities in anterior cervical spine surgery. HSS J. 2023;19(1):53–61. 10.1177/15563316221110572. 36776519 10.1177/15563316221110572 PMC9837402

[66] Agaronnik ND, Kwok A, Schoenfeld AJ, Lindvall C. Natural language processing for automated surveillance of intraoperative neuromonitoring in spine surgery. J Clin Neurosci. 2022;97:121–6. 10.1016/j.jocn.2022.01.015. 35093791 10.1016/j.jocn.2022.01.015

[67] Farber SH, Rudy RF, Zhou JJ, Alan N, DiDomenico JD, O’Neill LK, et al. Anatomical location of the bowel in different surgical positions: implications for lateral access in prone single-position surgery. Spine. 2025. 10.1097/brs.0000000000005272. 39876603 10.1097/BRS.0000000000005272

[68] Dodo Y, Okano I, Kelly NA, Haffer H, Muellner M, Chiapparelli E, et al. The anatomical positioning change of retroperitoneal organs in prone and lateral position: an assessment for single-prone position lateral lumbar surgery. Eur Spine J. 2023;32(6):2003–11. 10.1007/s00586-023-07738-w. 37140640 10.1007/s00586-023-07738-w

[69] Ouchida J, Kanemura T, Satake K, Nakashima H, Segi N. Anatomic evaluation of retroperitoneal organs for lateral approach surgery: a prospective imaging study using computed tomography in the lateral decubitus position. Eur Spine J. 2019;28(4):835–41. 10.1007/s00586-018-5803-x. 30377807 10.1007/s00586-018-5803-x

[70] Menezes CM, Andrade LM, Lacerda GC, Salomão MM, Freeborn MT, Thomas JA. Intra-abdominal content movement in prone versus lateral decubitus position lateral lumbar interbody fusion (LLIF). Spine. 2024;49(6):426–31. 10.1097/brs.0000000000004914. 38173254 10.1097/BRS.0000000000004914

[71] Mobbs RJ, Phan K, Malham G, Seex K, Rao PJ. Lumbar interbody fusion: techniques, indications and comparison of interbody fusion options including PLIF, TLIF, MI-TLIF, OLIF/ATP LLIF and ALIF. J Spine Surg. 2015;1(1):2–18. 10.3978/j.issn.2414-469X.2015.10.05. 27683674 10.3978/j.issn.2414-469X.2015.10.05 PMC5039869

[72] Smith TG, Pollina J, Joseph SA Jr, Howell KM. Effects of surgical positioning on L4–L5 accessibility and lumbar lordosis in lateral transpsoas lumbar interbody fusion: a comparison of prone and lateral decubitus in asymptomatic adults. World Neurosurg. 2021;149:e705–13. 10.1016/j.wneu.2021.01.113. 33548538 10.1016/j.wneu.2021.01.113

[73] Pimenta L, Amaral R, Taylor W, Tohmeh A, Pokorny G, Rodrigues R, et al. The prone transpsoas technique: preliminary radiographic results of a multicenter experience. Eur Spine J. 2021;30(1):108–13. 10.1007/s00586-020-06471-y. 32472346 10.1007/s00586-020-06471-y

[74] Ahlquist S, Park HY, Gatto J, Shamie AN, Park DY. Does approach matter? A comparative radiographic analysis of spinopelvic parameters in single-level lumbar fusion. Spine J. 2018;18(11):1999–2008. 10.1016/j.spinee.2018.03.014. 29631061 10.1016/j.spinee.2018.03.014

