# Supplementary material for: Influence of prone, supine, and lateral positions during spine surgery on vascular, abdominal, and postural anatomy: a comprehensive review and Bayesian meta-analysis
Source: Eur J Med Res. 2025 Oct 7;30:932. doi: 10.1186/s40001-025-03239-2 (PMC12502251; doi:10.1186/s40001-025-03239-2)
Supplement: Supplementary file 1 — Supplementary material 1. [file 40001_2025_3239_MOESM1_ESM.docx]

**Supplementary** **File** **1. Quantitative positional anatomy of the lumbar spine – individual‐study dataset supporting the meta‑analysis.**

**Contents**

1. Rationale and compilation methods
2. Complete comparison‑level table
3. Notes on data harmonisation and units
4. Abbreviation list

**1. Rationale and compilation methods**

We extracted the raw means ± standard deviations (SD) reported in all nine eligible studies (41 independent comparisons, 1 248 participants). Extraction was performed in duplicate (A.D., S.S.) using a piloted spreadsheet (Microsoft Excel 365). When results were plotted graphically, values were digitised with WebPlotDigitizer v4.6. Disagreements > 0.5 mm or 0.5° were resolved by consensus.

**2. Comparison‑level table has been provided on the next page.**


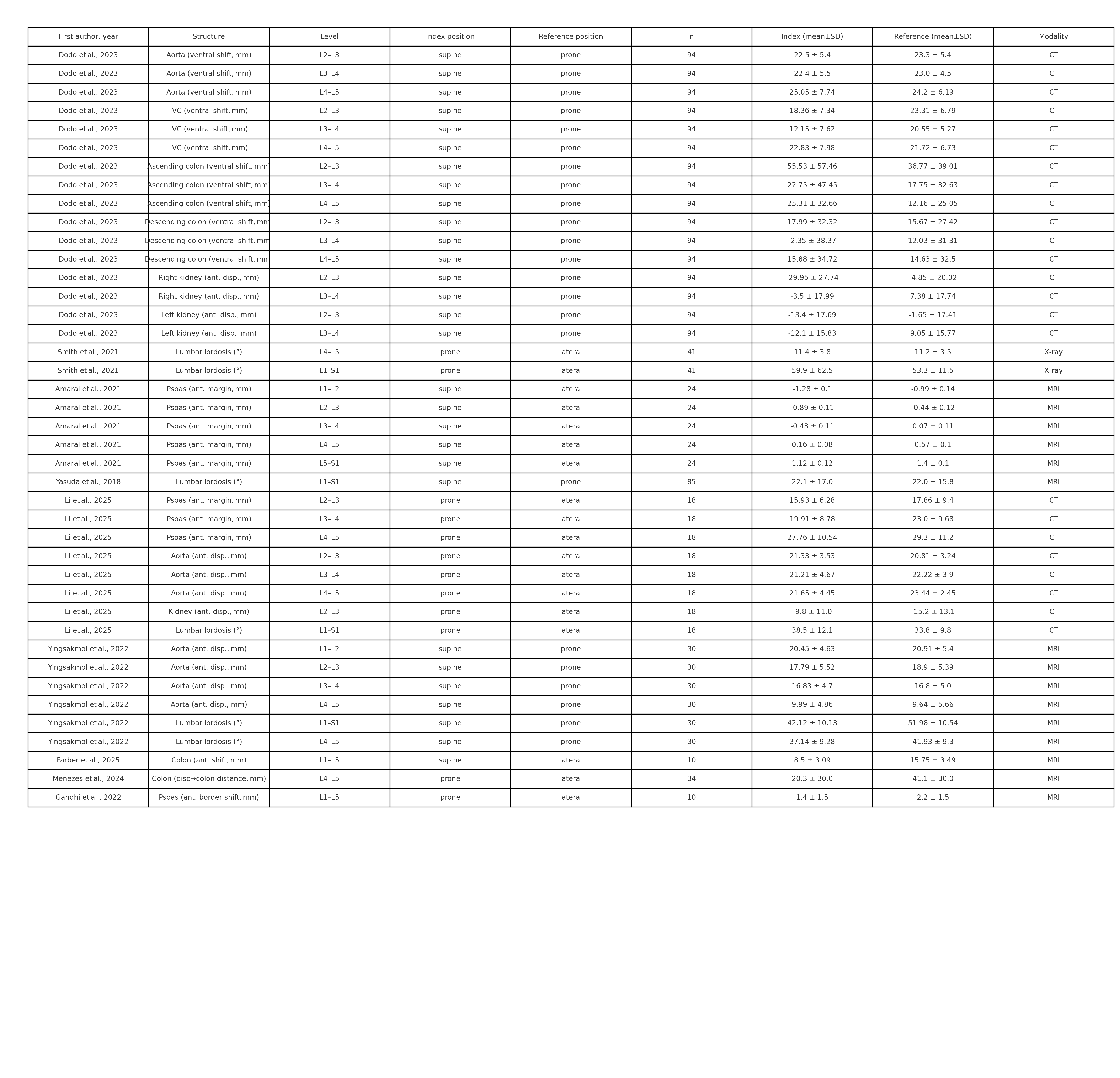
**4  Data harmonisation**

Units – All studies reported millimetres or degrees; no conversions were necessary.

Segmental lordosis – When only Cobb angles at adjacent endplates were given, the authors’ means and SDs were used directly; no back‑calculation was required.

Multiple levels within a study – Treated as separate comparisons but clustered under the same study identifier; variance–covariance was modelled using the robust (cluster) option in metafor.

Missing SDs – None; all nine studies provided measures of spread.
